# Sporadic colorectal cancer in adolescents and young adults: a scoping review of a growing healthcare concern

**DOI:** 10.1007/s00384-020-03660-5

**Published:** 2020-06-15

**Authors:** Natasha Christodoulides, Mariam Lami, George Malietzis, Shahnawaz Rasheed, Paris Tekkis, Christos Kontovounisios

**Affiliations:** 1 Chelsea and Westminster Hospital, London, UK; 2 Department of Surgery and Cancer, Imperial College, South Kensington Campus, London, SW7 2AZ UK; 3 Royal Marsden Hospital, London, UK

**Keywords:** Colorectal cancer, Sporadic, Young adults, Adolescents

## Abstract

**Purpose:**

Sporadic colorectal cancer (CRC) amongst adolescents and young adults (AYA) is increasing in incidence. The reasons for this trend are not well understood. Current guidelines do not specifically address this patient cohort. A scoping review was performed to summarise the range of available evidence and identify key areas that need to be addressed in current guidelines.

**Methods:**

A systematic literature search was conducted adhering to the PRISMA statement. All potentially eligible studies were screened, and data extraction was performed by two reviewers independently. The studies were then divided into 5 broad subgroups: (1) risk factors, (2) screening, (3) clinicopathological and molecular features, (4) presentation and (5) management. Descriptive statistics were used for data analysis.

**Results:**

A total of 17 studies were included from 2010 to 2019. Overall, young adults with CRC tend to present with non-specific symptoms. The majority of these patients have a delayed diagnosis and more advanced disease at presentation, with a rise in prevalence of distal colon and rectal cancers. AYAs tend to have poorly differentiated tumours and are managed more aggressively. Overall 5-year survival varies between studies.

**Conclusion:**

This is, to our knowledge, the first scoping review presenting the range of available evidence on CRC in AYAs. Although the rise in incidence is recognised by specialist bodies, recommendations are limited by the sparsity of available data. We seek to highlight the need for further research, define the role of earlier screening and raise awareness to promote thorough assessment of young patients.

## Introduction

Colorectal cancer (CRC) is the 2nd most common cause of cancer death in the UK and the 3rd most common cancer worldwide [1, 2]. Although CRC still remains a disease of older adults, incidence has been increasing in recent years amongst adolescents and young adults (AYAs) aged ≤ 49 years old [3, 4]. Although CRC linked to familial syndromes is more common in AYAs, the majority of cases in this age group are sporadic [5]. This patient group tends to have a delayed diagnosis and more advanced disease at presentation [3, 6, 7].

Recent research has identified the growing threat of CRC in AYAs through assessment of epidemiology, risk factors, molecular features and prognosis. To date, results have been insufficient to achieve an effective change in guidelines [8]. A scoping review was performed to summarise the range of available evidence and identify key areas that need to be addressed in current guidelines. Our primary objective is to present an overview of the available evidence pertaining to risk factors, screening, pathological and molecular features, presentation and management of CRC in AYAs. Our secondary objective is to review current guidelines addressing this patient cohort and make suggestions that can be incorporated into these guidelines.

## Methods

### Search strategy

We followed the scoping review framework proposed by Arksey and O’Malley, addressing all six stages of the methodological framework [9]. A comprehensive literature search was performed in consultation with a trained librarian, to identify all publications discussing the aetiology, screening, molecular features, presentation and management of colorectal cancer in AYAs. We reviewed the literature using MEDLINE, PubMed and EMBASE databases, in accordance with the PRISMA guidelines [10]. Keywords used in the search were: “colorectal cancer”, “under 50”, “colorectal neoplasms”, “pathology”, “young adult”, “United Kingdom”, “aggression”, “risk factor”, “screening”, “chemotherapy”, “diagnosis”, “surgery”, “epidemiology”, “management” and “practice guideline”. These keywords were selected to identify the maximum number of articles relevant to the chosen topic. All searches were performed in December 2019. No study limitations or time restrictions were set.

### Eligibility criteria

Articles were included in this scoping review if they met the following broad eligibility criteria: (1) published in English; (2) published in full-text format; (3) focussed on sporadic CRC in AYAs ≤ 49 years old; (4) discussed one of the following areas: risk factors, screening, molecular and pathological features, presentation and management; (5) level I to V evidence; (6) excluded articles discussing hereditary CRC syndromes only; (7) excluded other cancer types.

### Article selection and data extraction

The abstracts identified in the search were reviewed independently by two reviewers. Any disagreement between reviewers about whether a paper should be included resulted in inclusion at this stage in the process. The full-text articles were then reviewed by two reviewers and a final consensus was reached.

The studies were then divided into 5 broad subgroups: (1) risk factors; (2) screening; (3) clinicopathological and molecular features; (4) presentation and (5) management. Data relating to study characteristics (location of research, year, journal of publication, study design) was extracted, as well as data relating to one or more of the outcome themes (risk factors, recommendations for screening, unique pathological features, presentation and management).

### Data analysis

The data was summarised using descriptive statistics. Where applicable, data was analysed and presented as a proportion of the total. Categorical variables are reported as percentages. Inferential statistical testing was not performed. Level of evidence was assessed by assigning an Oxford Centre for Evidence Based Medicine (OCEMB) score to each paper [11].

## Results

**Fig. 1:**
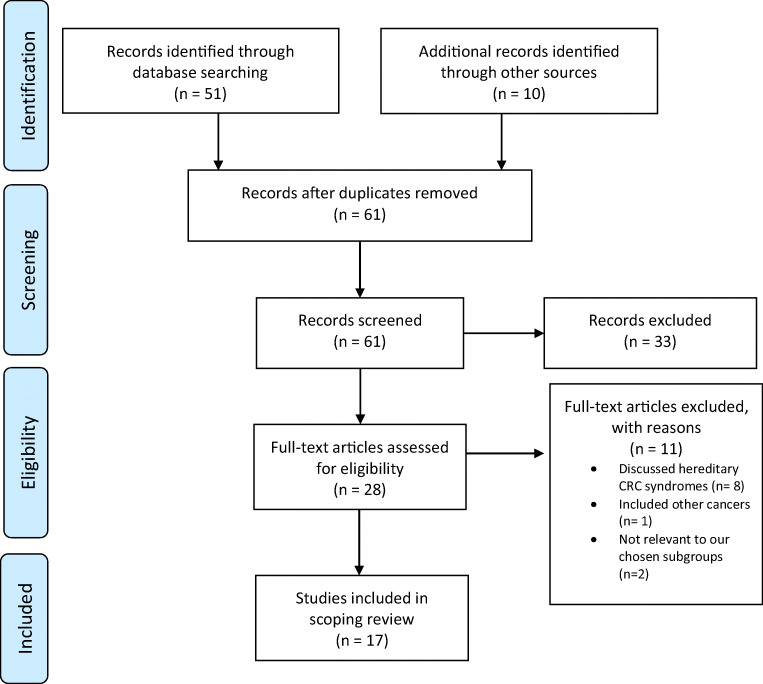
PRISMA 2009 flow diagram [10]

In total, 61 articles were reviewed, of which 44 were excluded due to the following reasons: the cut-off age for the young patient cohort was ≥ 50 years old, looked at other cancers, included hereditary syndromes only, were not relevant to our chosen subgroups (Fig. 1).

Our scoping review identified 17 papers from 2010 to 2019. The majority of included studies were from North America (52.9%), with some representation from Europe (23.5%) and Asia (23.5%). No relevant studies were published before 2010. A total of 88.2% of included studies were assigned an OCEMB score of 2 (Table 1). A minority of studies included patients in the ≤ 20 or < 50 age groups, whilst 82.4% of studies discussed patients in the ≤ 30 group and 58.8% included patients who were ≤ 40 years of age. A total of 94.1% of relevant studies discussed CRC, whilst one paper analysed and discussed trends in rectal cancer only. Study design and characteristics of the included studies are illustrated in Table 1.

**Table 1:** Study design and characteristics of the included studies

Characteristic	Frequency N (%) N = 17
Location of Research^a^:	
USA	8 (47.1)
Taiwan	2 (11.8)
Germany	1 (5.9)
Korea	1 (5.9)
The Netherlands	1 (5.9)
Canada	1 (5.9)
Italy	1 (5.9)
China	1 (5.9)
Czech Republic	1 (5.9)
Lebanon	1 (5.9)
Year of publication:	
2010–2014	7 (41.2)
2015–2019	10 (58.8)
Study design:	
Retrospective cohort study	13 (76.5)
Prospective comparative study	2 (11.8)
Case series	1 (5.9)
Letter	1 (5.9)
OCEBM Score:	
Level 2	15 (88.2)
Level 4	1 (5.9)
Level 5	1 (5.9)
Age of AYAs (years)^b^:	
≤ 20	4 (23.5)
≤ 30	14 (82.4)
≤ 40	10 (58.8)
< 50	2 (11.8)
Site:	
Colorectal	16 (94.1)
Rectal cancer	1 (5.9)
Outcome themes^c^:	
Risk factors	7 (41.2)
Screening	6 (35.3)
Clinicopathological and molecular features	4 (23.5)
Presentation	3 (17.6)
Management	6 (35.3)

^a^1 study was conducted in multiple locations

^b^Overlap between studies; 1 study not included as not applicable

^c^Some studies applicable to multiple outcome themes

### Risk factors

We identified seven studies which analysed and discussed risk factors for young-onset colorectal cancer. Recognised risk factors for CRC such as family history, lifestyle factors, metabolic syndrome and inflammatory bowel disease (IBD) were reflected in our results (Table 2).

**Table 2:** Risk factors identified in young-onset colorectal cancers

Authors	Country	Study design	Family history	Male gender	Race	Obesity/sedentary lifestyle	Unhealthy dietary pattern/processed meat	Smoking	Alcohol	Diabetes mellitus	Microbiota/IBD
Siegel et al. [12]	USA	Retrospective cohort study	–	–	–	Yes	Yes	–	–	–	–
Kwak et al. [13]	Korea	Prospective study	–	–	–	–	–	Yes	Yes	–	–
Chen et al. [14]	Taiwan	Retrospective cohort study	Yes	Yes	–	Yes	–	–	–	Yes	–
Kim et al. [15]	Korea	Cross-sectional analysis	–	Yes	–	Yes	Yes	Yes	Yes	Yes	–
Al-Barrak and Gill [16]	Canada	Retrospective cohort study	Yes	–	–	–	–	–	–	–	Yes
Rosato et al. [17]	Italy	Retrospective cohort study	Yes	–	–	No association	Yes	–	Yes	No association	–
Tawadros et al. [18]	USA	Retrospective cohort study	–	–	Yes^a^	–	–	–	–	–	–

^a^Race “other than black or white” identified as an independent risk factor

As highlighted by Weinberg et al., most young patients with CRC have sporadic disease, and only a small proportion of patients with familial CRC have an identifiable hereditary syndrome [5, 19]. Family history is recognised as a potential risk factor in 42.9% (*n* = 3) of the included studies. Al-Barrak and Gill, however, go one step further to suggest that family history of a first degree relative is less likely to be a factor in this patient population as, by virtue of age, family history is vigorously pursued in these patients [16]. In their retrospective cohort study, Tawadros et al. identified race “other than black or white” as an independent risk factor for rectal cancer in patients < 40 years of age [18]. Rosato et al. report no significant association between obesity and a sedentary lifestyle to CRC in subjects ≤ 45 years, although this is likely attributed to the small sample size [17].

Al-Barrak and Gill evaluated the role of inflammatory conditions in AYA CRC [16]. In this study, IBD was documented as a risk factor in 15% of patients ≤ 30 years old. These patients were more likely to present with metastatic disease (29% compared to 9% with localised disease) [16]. This is especially significant as IBD-associated dysplastic lesions have been shown to be more genomically unstable, perhaps reflecting a faster progression towards cancer and greater metastatic potential [16, 20].

### Screening

All 6 studies (100%) included in this subgroup recommended the consideration of earlier screening (Table 3). Following a retrospective analysis of incidence statistics from the Surveillance, Epidemiology and End Results (SEER) database, Davis et al. recommend colonoscopic age-based screening for average risk patients from 40 years [4]. Here, CRC locations were compared to determine the most suitable screening method [4]. Schellerer et al., however, recommend rigid rectoscopy from the age of 40, with colonoscopy or sigmoidoscopy with FOB testing in selected patients only [21]. This is based on evidence that two-thirds of young patients present with rectal tumours [21]. They suggest that lowering the age of screening from 50 to 40 would raise costs whilst identifying only 10% of CRC patients [21].

**Table 3:** Colorectal cancer screening

Authors	Country	Study design	Earlier screening recommended	Screening modality recommended
Davis et al. [4]	USA	Retrospective cohort study	Yes	Colonoscopic screening from 40 years
Schellerer et al. [21]	Germany	Letter	Yes	Rigid rectoscopy from 40 years
Siegel et al. [12]	USA	Retrospective cohort study	Yes	–
Singh et al. [3]	USA	Retrospective cohort study	Yes	–
Peterse et al. [22]	The Netherlands/USA	Retrospective cohort study	Yes	Colonoscopy every 10 years or FIT yearly or flexible sigmoidoscopy or CTC every 5 years from 45 to 75 years
Chang et al. [23]	USA	Retrospective cohort study	Yes	–

*FIT* faecal immunochemical test, *CTC* CT-colonography

In their retrospective cohort study, Peterse et al. describe the use of an adjusted Microsimulation Screening Analysis-Colon (MISCAN-Colon) model to make screening recommendations based on life-years gained [22]. They conclude that initiation of screening at age 45 offers a favourable balance between screening benefits and burden [22]. They advise either colonoscopy every 10 years, faecal immunochemical testing (FIT) every year or flexible sigmoidoscopy or CT-colonography every 5 years from 45 to 75 years of age [22].

### Clinicopathologic and molecular characteristics

We identified 4 papers discussing clinicopathologic and molecular characteristics of sporadic CRC in patients ≤ 49 years old. The age range of AYAs across studies was 14 to 45 years, with the percentage incidence approximately equal between genders (Table 4).

**Table 4:** Patient demographics and tumour characteristics

Authors and year	Country	Study design (no. of patients)		No. of patients		Time period	Age range young adults	% male, young adults	% male, older adults	Site	Most common site at diagnosis	Most common stage at diagnosis in AYAs	Survival, months	
Tawadros et al. [18]	USA	Retrospective cohort study	Multicentre (using SEER database)	*< 40*	*≥ 40*	2004–2010	20–39	56.2	59.6	Rectal only	N/A	Stage IIIb: 20.7% Stage IV: 18.4%	*< 40*	*≥ 40*
				1274	37,077								28 (median)	31 (median)
Chang et al. [23]	USA	Retrospective cohort study	Single centre	*≤ 40*	*> 40*	2000–2010	14–40	53	41	Colorectal—80% rectosigmoid in young adults	Left colon and rectum: 80% (*n* = 44)	Stage III: 36% (*n* = 20)	*≤ 40*	*> 40*
				55	73								53 (5-year survival)	57 (5-year survival)
Chou et al. [6]	China	Prospective cohort study	Single centre	*≤ 40*	*≥ 80*	2001–2006	22–40	47.8	79.8	Colorectal—52.3% rectosigmoid in young adults	Rectosigmoid: 52.3% (*n* = 36)	Stage III: 40.6% (*n* = 28) Stage IV: 42.0% (*n* = 29)	*≤ 40*	*≥ 80*
				69	253								44.1 (5-year survival)	51.0 (5-year survival)
Kothari et al. [24]	USA	Retrospective cohort study	Multicentre	*≤ 45*	*≥ 65*	1998–2010	30–45	55	49	Colorectal	–	Stage IV: 45% (*n* = 23)	N/A	
				51	195									

Italicized text represents the age cohort addressed in each individual study

The most common site at diagnosis in the younger age group was the left colon and rectum [6, 23] (Table 4). On review of tumour staging and prognosis, we identified that younger patients were more likely to present with stage III or IV CRC. Survival figures between the younger and older patient cohorts within each study were approximately equal. Survival varied between studies (Table 4).

Overall, on comparison of pathological features, younger patients were more likely to have poorly differentiated tumours, present with metastases and have cancers that are mucinous or signet-ring cell in histology (Table 5). Chang et al., however, identified no difference between the two groups when comparing mucinous histology [23]. Kothari et al. performed targeted exome sequencing to investigate whether genetic differences exist between age cohorts and found that mutations in the FBXW7 gene were more common in the younger group, as were mutations in the POLE proofreading domain [24].

**Table 5:** Analysis of pathological features

Authors	Mucinous		Signet-ring cell		Poorly differentiated		Metastasis	
	AYA %	Control %	AYA %	Control %	AYA %	Control %	AYA %	Control %
Chou et al. [6]	14.5 (mucinous or signet-ring cell)	6.3 (mucinous or signet-ring cell)	–	–	26.1	16.3	42.0	13.4
Chang et al. [23]	24	85	87	99	22.2	14.7	27	14
Tawadros et al. [18]	9.20	5.6	3	0.87	21.1	13.3	18.4	12.3

### Presentation

We identified 3 papers that analysed and reported data on the presenting symptoms of CRC in AYAs (Table 6). Rectal bleeding was the commonest symptom across all disease stages. Patients with stage IV disease commonly presented with rectal bleeding and abdominal pain [16]. Other common symptoms included change in bowel habit and tenesmus (Table 6). Reported symptoms are often non-specific and in the absence of risk factors can lead to delayed diagnosis [6, 7, 16]. Kocian et al. report that 78.9% of patients under the age of 40 presented with advanced-stage disease with resultant poor prognosis [7].

**Table 6:** Presenting symptoms

Authors	Country	Study design	No. of patients	Bleeding *n* (%)	Abdominal pain *n* (%)	Change in bowel habit *n* (%)	Tenesmus *n* (%)
Kocian et al. [7]	Czech Republic	Retrospective cohort study	38	18 (47.4)	7 (18.4)	8 (21.0)	4 (10.5)
Al-Barrak et al. [16]	Canada	Retrospective cohort study	45 (localised disease)	24 (53)	22 (49)	6 (13)	–
			17 (stage IV disease)	8 (47)	9 (53)	5 (29)	–
Chou et al. [6]	Taiwan	Retrospective cohort study	69	31 (28.7)	27 (25.0)	10 (9.3)	5 (4.6)

### Management

Six studies analysed the management of CRCs in AYAs (Table 7). Across these studies, the majority of cancers were located in the distal colon and rectum (Table 7). As discussed previously, young patients were more likely to present with advanced disease (TNM stage III or IV). Despite the advanced stage at presentation, the majority of young patients underwent surgical resection and chemotherapy. Compared to older CRC patients, aggressive treatment with multiagent chemotherapy regimens and biologics were more likely to be used in AYAs overall, including at earlier stages of disease [7, 26]. No significant difference however is reported in overall 5-year survival between the older and younger patient cohorts [6, 7, 26]. Zhao et al. report significantly lower 5-year survival in the younger group with stage III disease [27].

**Table 7:** Management of CRC in AYAs

Authors	Country	Study design	Tumour types (% patients)	No. of patients	TNM staging (% patients)	Surgery (% patients, type)	Chemotherapy rates (% patients)	5-year overall survival (% patients)
Kocian et al. [7]	Czech Republic	Retrospective study	60 rectum, 40 colon	38	I 20 II, III and IV- 80	82 (curative + palliative)	100	47.9
Farraj et al. [25]	Lebanon	Case series	52 rectum, 45 colon	29	–	–	62	–
Al-Barrak et al. [16]	Canada	Retrospective study	36 rectum, 64 colon	78	I 9 II 42 III 49	76 (primary resection)	–	12
Kneuertz et al. [26]	USA	Retrospective study	Relative to splenic flexure: Distal 56.3, proximal 39.9	13,102	I 14.7 II 23.5 III 36.5 IV 25.3	100	66.8 (single agent 15.6, multiple agent 42.8, unknown 7.8)	61.7
Chou et al. [6]	Taiwan	Retrospective study	52.6 rectosigmoid; 47.7 colon	69	I 8.7 II 8.7 II 40.6 IV 42	87 (radical resection)	83	44.1
Zhao et al. [27]	China	Retrospective study	44.1 rectum; 55.9 colon	68	N/A (used AJCC)	100	85.3	66.4

## Discussion

Seventeen studies across 10 countries evaluating risk factors, presentation, screening, management and clinicopathological and molecular features of sporadic young-onset colorectal cancer were included. Whilst other narrative reviews have been published [8, 28], this is to our knowledge the first scoping review presenting the broad range of available evidence.

Although the absolute incidence of colon and rectal cancers in young adults ≤ 49 years of age is low overall, a change in epidemiology with a significant rise in incidence has been clearly documented in the literature [3, 5, 29]. A recently published systematic review by Saad El Din et al., assessing population trends of CRC in AYAs, highlights the increasing risk of CRC in this patient cohort, largely driven by rectal cancers [29]. Current guidelines do not reflect this epidemiological shift, which poses new challenges in screening, diagnosis and management.

The National Comprehensive Cancer Network (NCCN) guidelines recognise the rise in incidence of sporadic CRC in the young [30]. It is estimated that the incidence rates for colon and rectal cancers will increase by 90% and 124.2% respectively for patients 20–34 years old by 2030 [30]. Moreover, if it becomes broadly accepted that CRC in the young is a unique disease entity, there would be a need to develop targeted treatment strategies [30]. A summary of the relevant information available in guidelines from national and international specialist bodies is highlighted in Table 8.

**Table 8:** Summary of age-specific recommendations for the management of CRC in AYAs, from national and international guidelines

NCCN [30]	• Management recommendations are made based on tumour stage and high-risk features
	• Overall benefit and toxicities of 5-FU/LVAS adjuvant therapy is similar in older and younger patients
	• Advise consideration of more extensive colectomy for patients < 50 years old
NBOCA [31]	• Younger patients are more likely to receive chemotherapy
	• Younger patients are more likely to have long-course radiotherapy
NICE [32]	• No specific recommendations for young patients ≤ 49 years old
ESMO [33]	• No specific recommendations for young patients ≤ 49 years old
ACS [34]	• Regular screening at the age of 45 in people at average risk of CRC
	• Patients at “average risk” should not have: a personal of family history of CRC, a personal history of IBD, a confirmed or suspected hereditary CRC syndrome, a personal history of radiotherapy to the abdomen or pelvis
	• Testing options: stool based (FIT yearly or FOB yearly or MT-sDNA 3 yearly); structural examinations (colonoscopy 10 yearly or CTC 5 yearly or flexible sigmoidoscopy 5 yearly)

*NCCN* National Comprehensive Cancer Network, *NBOCA* National Bowel Cancer Audit, *NICE* National Institute for Health and Care Excellence, *ESMO* European Society of Medical Oncology, *ACS* American Cancer Society, *FIT* faecal immunochemical test, *MT-sDNA* multi-target stool DNA, *CTC* CT-colonography

All studies included under the outcome theme of screening recommend that earlier screening should be instigated (Table 3). Currently, CRC screening in the UK in patients with no known risk factors begins at the age of 60 with a faecal occult blood test (FOBT) [32]. Screening occurs every 2 years up to the age of 74. In some areas, a one-off colonoscopy is offered at the age of 55 [32]. The American Cancer Society (ACS) has updated its guideline on colorectal cancer screening and now recommends screening at 45 years of age for patients at average risk [34]. In a UK population-based analysis, Yong et al. demonstrate the rising incidence of CRC in AYAs [35]. While a cost-effectiveness analysis needs to be carried out before any changes can be made to UK National Bowel Cancer screening guidelines, based on our scoping review, we propose screening with a flexible sigmoidoscopy at the age of 40 in patients at average risk. To do this successfully, it is essential to raise awareness, particularly in primary care. We propose developing questionnaires which incorporate known risk factors, to be used opportunistically in the community to identify patients ≥ 40 years old who are at average risk. To successfully define “average risk” in the UK, further population-based studies are required. With recall systems already in place in primary care, implementation of earlier screening could be feasible.

The National Institute for Health and Care Excellence (NICE) guidelines do not report any specific recommendations for management of sporadic colorectal cancer in the young [32]. With regard to the management of young CRCs, we have very limited data on the types of resections performed and outcomes after chemotherapy to make robust recommendations. The National Bowel Cancer Audit (NBOCA) states that in the UK, patients who are younger with fewer comorbidities (ASA 1/2 and performance status 0/1) are more likely to receive chemotherapy [31]. In addition, younger patients are more likely to have long-course radiotherapy [31]. There is little evidence on the effectiveness of this aggressive management approach with guidelines for treatment based largely on older adults [5] . The better overall physical status of younger patients may mean that clinicians not only opt to treat this group more aggressively, but also that patients are more resilient to these treatment strategies. However, adopting an aggressive approach to treatment of AYAs, based on minimal research, may result in significant long-term effects and a poor quality of life with little survival benefit [26].

Following a review of the clinicopathologic characteristics presented, we hypothesise that CRCs in AYAs comprise a combination of a more aggressive and unique biological nature of tumour (Table 5). The genetic differences between age cohorts reveal the need for alternative treatment strategies and particular attention from oncology [19], specifically in the delivery of targeted therapy. The European Society for Medical Oncology (ESMO) guidelines recommend applying personalised medicine in the management of high-stage CRC [33]. Overall, further research is needed to target CRC in the young and develop specific treatment plans that reduce recurrence, increase overall survival and have the least detrimental long-term effect.

The majority of young adults with CRC across the included studies presented with rectal bleeding. The vague presentation of this disease requires increased awareness amongst clinicians, thus avoiding delayed diagnosis which may result in more advanced disease at presentation [35].

Research into biomarkers specific to sporadic CRC in the young is necessary to aid molecular subtyping of this disease entity, allowing earlier detection and personalised management. To further research, we suggest that a serum sample and all tumours from younger patients with CRC are sent for genetic testing [28]. This would promote expansion of the gene pool and perhaps allow collaborative research efforts into the pathological features of early onset sporadic CRCs.

### Limitations

Our literature search was limited to studies published in English, which may have affected the inclusion of a number of potentially eligible studies from non-English speaking countries. The limited number and heterogeneity between studies meant that a systematic review was not feasible. Additionally, the variability between studies introduced challenges in presenting details of all study data.

## Conclusion

Sporadic CRC in AYAs is a growing health concern that is underrepresented in clinical research [5]. Although recognised by national and international bodies, recommendations are limited by the sparsity of available data. In this scoping review, we have presented a comprehensive summary of available evidence based on five broad themes. The heterogeneity of the included studies presents challenges to making robust recommendations that can be incorporated into current guidelines. Given the rising incidence of this disease, we seek to highlight the need for further research aimed towards defining the role of earlier screening. Particularly, given its more aggressive histology, we seek to raise awareness and promote thorough assessment of young adults presenting with colonic and rectal symptoms.

## Data Availability

All data is readily available.
